# Patient satisfaction with peri-operative anesthesia care and associated factors at two National Referral Hospitals: a cross sectional study in Eritrea

**DOI:** 10.1186/s12913-019-4499-x

**Published:** 2019-09-18

**Authors:** Yonatan Mehari Andemeskel, Traudl Elsholz, Ghidey Gebreyohannes, Eyasu H. Tesfamariam

**Affiliations:** 1 Department of Anesthesia and Critical Care, School of Nursing, Asmara College of Health Sciences, Asmara, Eritrea; 2 Asmara College of Health Sciences, Asmara, Eritrea; 3 Department of Epidemiology and Biostatistics, School of Public Health, Asmara College of Health Sciences, Asmara, Eritrea

**Keywords:** Patient satisfaction, Perioperative care, Leiden preoperative care patient satisfaction questionnaire (LPPSq), Information provision, Staff-patient relationship, Fear and concern, Anaesthesia

## Abstract

**Background:**

Measuring patient satisfaction has become an important parameter of the continuous quality assessment and improvement in anaesthesia services. The aim of this study was to assess the level of patient satisfaction with perioperative anaesthesia care and to determine the factors that influence satisfaction.

**Method:**

This study is an cross sectional design, conducted on 470 patients who underwent different types of surgeries at two National Referral Hospitals in Asmara, Eritrea between January and March of 2018. Patients were interviewed 24 h after the operation using a Tigrigna translated Leiden Perioperative Care Patient Satisfaction questionnaire (LPPSq). Descriptive and inferential analysis were made using SPSS (version 22). Statistical significance level was set at *P <* 0.05.

**Results:**

The overall satisfaction score was 68.8%. Less fear and concern was observed among patients with satisfaction scores of 87.5%. Staff-patient relationship satisfaction score was 75%. Patients were least satisfied with information provision (45%). Multivariable analysis revealed that satisfaction of patients who did surgery at Halibet hospital is significantly higher (*p* < 0.001) than those patients who did at Orotta hospital. Moreover, those patients who did elective surgery had higher level of satisfaction that those who did emergency surgery (*p* < 0.001).

**Conclusion:**

Moderate level of satisfaction was observed among the patients. Generally, the study emphasized that the information provision about anesthesia and surgery was low. Patients described better staff-patient relationship and low fear and concern related to anesthesia and surgery was observed.

**Electronic supplementary material:**

The online version of this article (10.1186/s12913-019-4499-x) contains supplementary material, which is available to authorized users.

## Background

Patient satisfaction is a complex concept which highly depends on the subjective judgment of a patient. It is related to a number of factors including patient’s emotional, social, and cultural factors and values, past experiences, and future expectations [1–5]. It refers to the degree of fulfilment of patients’ expectations by the care provided [6]. Patients tend to compare their expectations with the experiences they had as well as with the actual outcomes. When those expectations are not met by the actual situation, the patient may become dissatisfied. Hence, patient’s satisfaction depends on the consistency between what the patient expects and what is actually perceived or experienced [4, 7, 8].

Patient satisfaction is also a component of quality of care. Enhancing the quality of service improves the satisfaction of patients [9–11]. Moreover, the quality of service and the satisfaction of customers are key determinants of patient’s loyalty [12]. As few patients have repeated surgery, it cannot be surely described whether it encourages for future service [11]. However, it is more likely for loyal customers to return for service and they would also recommend it to their families, friends and other service rendering customers [13]. Strengthening the communication skills with the patients is also among the important determinant factors of patient satisfaction [12]. It determines the adequacy of information provided during the preoperative period and having and empathic attitude towards the patient during this period can possibly decrease patient’s anxiety while increasing patient satisfaction and there by improves the quality of service [14]. Poor quality of anaesthesia services may discourage patient from using available services and as people are becoming more and more aware of their rights and what they can expect, the demands of best possible care is increasing even also in developing countries. Therefore, it remains the duty of every staff to deliver the best possible care. Consequently, many health care organizations have considered the measurement of patient satisfaction to be a critical component of quality assessment. Thus, nowadays assessment of patient satisfaction with anaesthesia services is a reality of practice [1, 9]. Having sufficient data related to patient satisfaction is assumed to improve and understand its strengths and to target areas in which performance is insufficient and it provides an opportunity for the improvement or change of the identified gaps [3, 9, 15–19]. Furthermore, the assessment of patient satisfaction with the care they experience is a key performance measure that is increasingly used in various payment models and payment for performance plans. In the future, it is likely that payment for anaesthesia services will depend in part on the degree of patient satisfaction [9].

In perioperative anaesthesia practice, the patient perspective on their care can be sought through the administration of patient satisfaction surveys [20]. No research has been conducted before related to patient satisfaction with the anaesthesia care in Eritrea. The aim of this study was to assess, analyze and evaluate the degree of patient satisfaction on perioperative anaesthesia care among Eritrean surgical patients and to identify factors associated with satisfaction. The study is also expected to be significant in providing information on patient satisfaction with anaesthesia care from patients’ point of view.

## Methods

### Study setting

Study was conducted at Halibet & Orotta national referral hospitals which are found in the Asmara, the capital city of Eritrea. Both hospitals provide health services at a tertiary level. They were selected because they are the only governmental medical surgical national referral hospitals where all types of major and minor surgeries take place.

### Study design

This cross sectional study was conducted between January and March of 2018. Data was collected by four trained surveyors through face to face interview using a stratified questionnaire.

### Sample

**Fig. 1:**
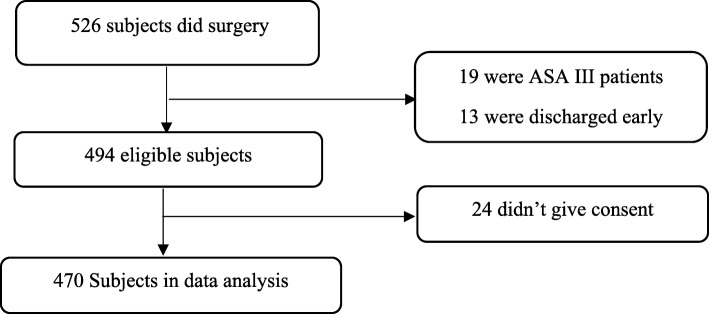
Number of patients eligible for the study, included in the study, and analysed

During the study period a total of 526 patients underwent surgeries under general and regional anaesthesia within the 3 month period. The eligibility to participate in the study was based on the respondent’s willingness to take part in the study. Patients under the age of 18, those who were discharged before 24 h of postoperative period, those who were seriously ill, and those who didn’t give consent were excluded from the study. Finally, 470 were found to fulfil the inclusion criteria and thus were included in data analysis. (Fig. 1).

### Questionnaire

The key elements of socio-demographic characteristics of the patients were obtained using a socio-demographic form. A validated English version Leiden Perioperative care Patient Satisfaction questionnaire (LPPSq) which was designed to measure the patient satisfaction with perioperative anesthsia service was adopted. This questionnaire is acceptable and suitable for research purposes [16]. Permission was asked and obtained from the responsible author. The questionnaire contains three aspects of the peri-operative evaluation. The first aspect is patients’ satisfaction which contain three dimensions (information, fear and concern, staff-patient relationship). The second aspect of the questionnaire assesses professional competence and service and the third one assesses the prevalence of undesirable anesthesia outcomes (discomfort and needs).

### Variable measurement

In order to measure satisfaction of the patients, items of information (*n* = 5), fear and concern (*n* = 4), and staff-patient relationship (*n* = 11) were used. Moreover, the professional competence and service were measured for exploratory purpose. However, the prevalence of perioperative undesirable anesthesia outcomes is not included here. Responses to questions of three dimensions of satisfaction, namely ‘information’, ‘fear and concern’, and ‘staff patient relationship’ were standardized to a five point Likert type scale. The replies to information and staff-patient relationship were “Completely satisfied”, “Satisfied”, “Neither satisfied nor dissatisfied”, “Dissatisfied” and “Completely dissatisfied”. Two questions from the ‘service’ dimension, which discuss about the waiting time were also having five point Likert scale. The replies to the dimension “fear and concern” are “Not at all”, “A little bit”, “Moderately” and “A little bit more” and “Extremely”. However, the replies to the rest of the items for “professional competence” and “service” dimensions were “Yes” and “No” only.

While developing and validating the questionnaire, the overall internal consistency of LPPSq was 0.90 with three identified dimensions, namely, information (Cronbach α = 0.82), fear and concern (Cronbach α = 0.69), and staff–patient relationship (Cronbach α = 0.94). The inter-item correlation (IIC) ranged from 0.53 to 0.83 [21]. In another study conducted to determine patient’s satisfaction, the reliability estimates of information (Cronbach α = 0.95), fear and concern (Cronbach α = 0.65), staff-patient relationship (Cronbach α = 0.98), and the total English LPPSq (Cronbach α = 0.94) were satisfactory. Moreover, the inter-item correlation ranged from 0.56 to 0.89 [22]. In our study, the content validity of the questionnaire was insured by expert opinions from the anaesthesia department. It was then translated to the dominant local language (Tigrigna) and back translated and was pretested for the purpose of assessing the comprehension and degree of understandability of the questions.

### Data analysis

Data was entered into SPSS (Version 22) statistical software for analysis. Data cleaning and preliminary explorations were done before conducting the main analysis. During the first stage, the reliability, validity and factor structure of the questionnaire were evaluated using exploratory factor analysis. Internal consistency of the satisfaction scale was assessed using Chronbach α. Item discriminant validity and inter-item correlations were computed using Spearman rank correlation coefficient. Inter-item correlations (ICC), which show strength of the relationship between items of the same dimension and item discriminant validity (IDV) which signifies the relationship between the items of different dimensions were computed. Chronbach alpha is expected to be greater than 0.7 [23]. Frequency (percent), mean (SD), median (IQR) were used to make descriptive analysis, as appropriate. The overall level of satisfaction and the level of satisfaction in each dimension were then obtained using the sum of the item responses converted into a percentage. After assessing the normality of overall satisfaction percent using Kolmogrov-Smirnov test, comparisons across categories of patient characteristics were done using independent samples t-test or one way ANOVA (LSD post hoc). Variables found to be significant at bivariate level were further analysed at multivariable level. Partial eta squared (percent of the satisfaction explained by the knowledge of the corresponding independent variable) and power were also computed. *P*-values less than 0.05 were considered as significant throughout the analysis.

## Results

### Population characteristics

Out of the 526 patients who did surgery, 470 subjects gave their consent and were eligible for data analysis. Of all the patients, 55.1% were males and 44.9% were females. The mean age was 45.9 ± 14.7 ranging from 18 to 85 years. The majority (63.2%) of the patients were from Orotta Hospital. The patients underwent a wide range of surgical procedures, including general, orthopaedic, Gyn/obs, ENT and burn surgery. 267 (56.8%) patients had general anaesthesia, and 203 (43.2%) regional anaesthesia. Almost all patients (96.8%) did major types of surgeries. Patient characterstics is available as Additional file 1.

### Psychometric property of the patient’s satisfaction tool

Before computing the satisfaction level of the patients, the construct validity of the subscales of satisfaction was presented. In addition, exploratory factor analysis was done and Chronbach Alpha for scale reliability was also computed.

### Exploratory factor analysis

Kaiser-Meyer-Olkin measure revealed that the factor structure analysis was appropriate (KMO = 0.847). The Bartlett Test of Sphericity was also significant (Chi-square = 4787.639, *p* < 0.001). Principal component analysis using varimax rotation identified three dimensions from all the items in the questionnaire that were developed to measure satisfaction. The identified dimensions were similar to the original item groups, namely, “information”, “fear and concern” and “staff-patient relationship”. Factor loadings and the percent variance explained by each component are presented in Table 1.

**Table 1:** Components identified, factor loading, and Chronbach’s alpha of LPPSq

Items	Factors		
	1	2	3
Operating room staff take into account your personal preferences	.861	–	–
Staff ability to understand for your situation	.842	–	–
Anaesthetist pay attention to you as an individual?	.815	–	–
Staff professionalism	.723	–	–
Staff pay attention to complaints like nausea & vomiting	.694	–	–
Pay attention to questions asked	.619	–	–
Confidence on the anaesthetist	.607	–	–
Staff attitude	.591	–	–
Quality of anaesthetic care given during operation	.560	–	–
OR staff take into account your privacy	.491	–	–
Information about anaesthesia during the preoperative period	–	.874	–
Information about post-operative feelings	–	.839	–
Information about surgery	–	.830	–
Information about duration of stay	–	.524	–
Information about fasting time	–	.507	–
Preoperative assessment done	.402	.440	–
Fear of seeing the operating room	–	–	.893
Fear of awakening during surgery	–	–	.857
Fear & Concern of pain due to surgery	–	–	.734
Fear & Concern due to Anaesthesia/anaesthetist	–	–	.567
Percent of Variance Explained (55.09%)	25.51	16.69	12.89

### Reliability

As shown in Table 2, the reliability estimates of information (Chronbach α = 0.808), fear and concern (Chronbach α = 0.782), and staff-patient relationship (Chronbach α = 0.882) were satisfactory. Correlations between the items and their dimensions (*inter-item correlation*) ranged from 0.438–0.887. The score for internal consistency for the items of all dimensions (*item discriminant validity*) was low indicating a weak correlation between the scores of items of each dimension with other dimensions.

**Table 2:** Psychometric properties and descriptive summaries of the three scale components and total LPPSq

Dimension	Number of items	Chronbach α	Median dimension score (IQR)	Maximum dimension score	Inter-item correlation (IIC)	Item-discriminant validity (IDV)
Information	5	0.808	9 (8)	20	0.462–0.887*	0.020–0.387; 0.408^a^
Fear & concern	4	0.782	14 (6)	16	0.438–0.882*	0.002–0.302
Staff-patient relationship	11	0.882	33 (0)	44	0.509–0.631*	0.002–0.387; 0.408^a^
LPPSq	20	0.763	55 (31)	80	†	†

† Cannot be computed, * Significant at *p* < 0.001, ^a^ only one item was found to have IDV > 0.4.

### Overall satisfaction

**Fig. 2:**
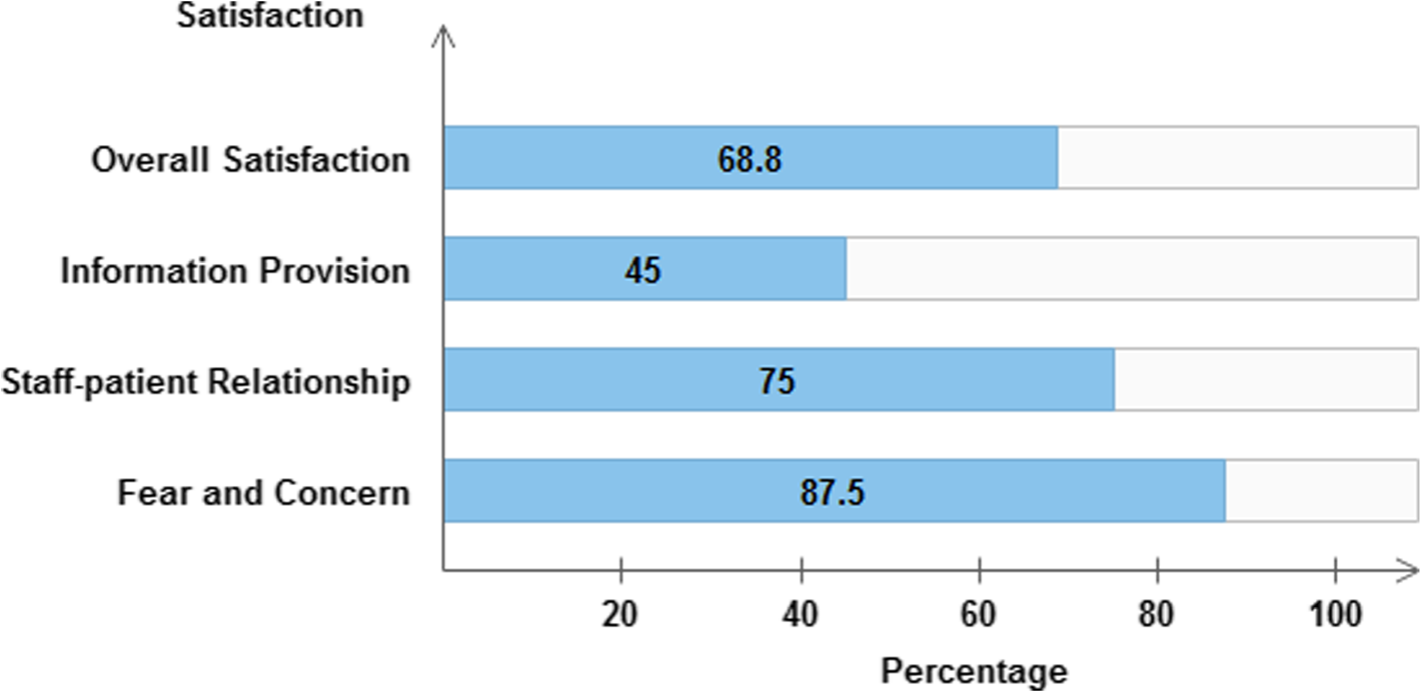
Overall and subscale percent patient satisfaction scores

The overall average satisfaction of patients with the perioperative anaesthesia service was 68.8%. When the dimension scores were compared with each other, lowest patient satisfaction score was for the dimension information provision (45%) and the component with the highest satisfaction score was for fear and concern (87.5%). The third dimension staff-patient relationship scored 75% of the satisfaction (Fig. 2).

### Association of Patient variables with the overall satisfaction

The overall satisfaction of patients was cross tabulated with patient demographic and clinical variables. Mean satisfaction score was found to increase with increase age (*p* = 0.033). Males were more satisfied than females (70.9 vs 67.8%, *p* = 0.001). Patients who came from urban settings were more satisfied compared to those who came from a rural setting (70.36% vs 68.28%, *p* = 0.033). Surgery was done in two different settings and those who did surgery in Halibet hospital were more satisfied (*p* < 0.001) with a mean satisfaction score of 73.7% as compared to those who did surgery in Orotta Hospital (67.0%). Type of anaesthesia also had a significant influence on the overall patient satisfaction (*p* = 0.011). Patients who took general anaesthesia were less satisfied (54.8%) than those who took regional anaesthesia (56.7%). Surgical specialty also had an effect on the level of patient satisfaction (*p* = 0.021). Satisfaction score ranged from 73.8% for orthopaedic surgeries to 66.1% for Gyn/Obs surgeries (*p* < 0.001). Patients who did elective surgeries were found to be more satisfied (*p* = 0.001) with a mean satisfaction score of 70.4% as compared to those who did emergency surgeries who scored 66.6% (Table 3).

**Table 3:** Satisfaction related socio-demographic and clinical characteristics of patients

Variable	Mean (SD)	*p*-value	*p*-trend
Gender			
Male	70.90 (11.04)	0.001	–
Female	67.77 (9.29)		
Age (r = 0.120, *p* = 0.009)			
18–35	67.70 (9.86)	0.023	0.011
36–60	70.26 (10.97)		
61–85	70.74 (9.84)		
Residence			
Urban	70.36 (11.04)	0.033	–
Rural	68.28 (9.31)		
Occupation			
Employed	71.29 (10.63)	< 0.001	–
Unemployed	67.37 (10.09)		
Hospital Setting			
Halibet	73.70 (12.37)	0.0001	–
Orotta	67.04 (8.13)		
Type of Anaesthesia			
General	54.75 (8.08)	0.011	–
Regional	56.70 (8.52)		
Type of Surgery			
General	69.01 (9.72)	< 0.001	GS < OS (0.011)
Orthopedic	73.81 (13.05)		GOS < OS (0.011)
Gyn/Obs	66.14 (7.52)		GS *≠* GOS (0.346)
Admission type			
Emergency	66.57 (10.10)	0.001	–
Elective	70.37 (10.34)		

*GS < OS=Satisfaction among General surgery patients is significantly less than Orthopedic surgery patients.*

*GOS < OS=Satisfaction among Gyn/Obs Surgery patients is significantly less than Orthopedic surgery patients.*

*GS≠OS=No significant difference in satisfaction between General surgery Patients and Gyn/Obs surgery patients.*

Multivariable analysis of the factors that affect satisfaction was performed by selecting variables that were found to be highly significant at the bivariate analysis (i.e. *p* < 0.01). At multivariable level, only the hospital at which surgery was done (*p* < 0.001) and admission type (*p* < 0.001) were found to be significant predictors of satisfaction (Table 4).

**Table 4:** Main predictors of patients’ satisfaction using multivariable analysis

Patient Variables	*F*-value	*p*-value	Partial Eta Squared	Observed Power^a^
Gender	2.56	0.111	0.006	0.358
Patient occupation	3.10	0.079	0.007	0.419
Hospital	17.91	< 0.001	0.041	0.988
Type of Anaesthesia	0.10	0.757	< 0.001	0.061
Surgery kind	0.04	0.961	< 0.001	0.056
Admission type	12.58	< 0.001	0.029	0.943

*Errors were found to be normally distributed after fitting the multivariable model.*

*R Squared = .148 (Adjusted R Squared = .134),* ^*a*^ *Computed using alpha = .05.*

### Professional competence and service

Most (85%) of the patients told that the anaesthetist didn’t introduce themselves to them and only half of the patients stated that enough information about anaesthesia was given to them. Most of the patients were satisfied with the fact that the anaesthetist listened (84%) and acted (89.8%) according to their needs. The overall median score of professional competence was 75%. Almost 80% of the patients were seen by the anaesthetist before the operation during the preoperative period. 86.2% of the patients underwent operation without delay on the agreed date and time. Most (93.4%) of the patients stated that they have a good understanding of the role of the anaesthetist and 95.7% explained that they would want to have the same anaesthesia again or they would recommend the same care or service to family and friends.

Waiting time was assessed as being long (scored as 0), enough (scored as 1) and short (scored as 2). The median was found to be two in both items, indicating that short waiting time was experienced in the majority of the patients.

## Discussion

This study was conducted under the main objective of assessing the level of satisfaction of patients on perioperative anaesthesia care. The internal consistency of the satisfaction components was good. These components address particularly the patients’ experience of care and satisfaction about the information, fear and concern, staff-patient relationship including the professional competence and the service provided.

The findings of the study revealed that the overall satisfaction level of the patients with peri-operative anaesthesia care at the described sites was 68.8%. This score was low compared to the findings of the two studies made by the adopted LPPSq questionnaire in which the overall mean satisfaction level in the Netherlands and England were 92.1 and 86.7% respectively [21, 22]. Studies in which a similar questionnaire (LPPSq) was used were also conducted in Saudi Arabia and Rwanda. The overall satisfaction of the Rwandan patients was similar to this study (67.3%) while the Saudi Arabian patients were a little less satisfied (61.9%) [24, 25]. A study done in Ethiopia that focused only on preoperative period revealed an overall satisfaction level of 65% [26].

The dimension information provision involves specific questions about the explanation and amount of information provided to patients regarding anesthesia, surgery, and their stay in the operating theatre. The overall patient satisfaction score was low in the area of information provision (45%). Such lowered scores could be associated with the fact that adequate information is not provided to the patients during the perioperative period especially related to the anaesthetic care. Sometimes pre-assessment was not done the day before the surgery and the patient was just quickly assessed before surgery in operating room. This could have reduced adequate time with the patient for the anaesthetist. Similarly, the component with the lowest score in the other similar researches was information provision [21, 22, 24, 25]. In the study made by Heidegger and colleagues, problems mentioned most were in the components ‘Information/Involvement in decision-making’ (mean problem score: 30.9%) [15]. Lack of preoperative surgical information and guidance was also one of the causes of dissatisfaction according to the study done at Sohag University [25]. Moreover, in an Ethiopian study, only 32.4% of the patients received information about the type of anaesthesia and only 20.6% of the patients received information about possible postoperative complications [26]. Preoperative communication from anaesthetists outlining anaesthesia options and realistic postoperative expectations cannot only alleviate anxiety, but offers patients a sense of control over their care. Healthcare providers should have the responsibility and opportunity to improve patient care through the adoption of standardized communication processes [27, 28].

In order to help to decrease situational anxiety, anesthetists can improve information exchange, by allowing sufficient time for patient questions and adopting explanations to patient needs [14]. Despite this fact, information provision was rated low in this study, even though the satisfaction for the component ‘fear and concern’ was considerably high (87.5%). Such a score was similar when compared to the previously mentioned studies [21, 22]. The findings were much higher when compared to the study done in Rwanda in which the mean satisfaction score of the dimension fear and concern was 57.3%. The dimension staff-patient relationship assesses the competency and professionalism of the operating theatre staff as perceived by patients, whether staff were attentive to patient needs, and act according to their needs. The percentage satisfaction score for time dimension was 75%. This score was low when compared to the results of Calijouw et al., (2008) and Jlala et al., (2010) in which the satisfaction scores were 92.1 and 90.3% respectively, but a little higher than in the Rwandan study (72.1%) [24].

In this study multivariable analysis revealed that the main predictors of patient’s satisfaction were hospital at which surgery was done and the admission type as well. As expected satisfaction was higher among those patients who did elective surgeries (*p* < 0.001), and this could be associated with the fact that patients, who had elective surgery, had more time to communicate with the anaesthetist.

### Professional competence and service

In this study, failure of the anesthetist to introduce himself or herself to the patient was one of the weaknesses noticed in this study. Only 15% introduced themselves to the patient. Such a weakness cannot be excused by it’s being a habit seen not only among the anaesthetists but among many other professionals as well. Although most of the anaesthetist were not able to introduce themselves, their abilities to listen and act according to the patients’ needs amazingly were highly satisfying (89.8%). In a study done in Ethiopia, self-introduction to anaesthetist was similarly low in which only 23.5% of the anaesthetist were able to introduce themselves to their patients [26]. The overall professional competence of the anaesthetists was rated by the patients as 75%. Higher professional competence was scored elsewhere [21, 22].

Healthcare professionals may be rated differently by patients. In this study, only 79% of the patients reported that they were seen by the anaesthetist on the day before operation. However, as the anaesthetists did not introduce themselves to the patients (81.9%), such a low score could also be associated with the inability to recognize the anaesthetist as such. The fact that 22.1% of the patients were not seen the day before operation is an issue of concern in the practice of anaesthesia. Regarding the waiting time, in some of the operating rooms the patients are made to come for operation all at the same time and this could increase the waiting time until they go in for the procedure. In reflection to that, 50% of the patients explained that the waiting time was long.

### Study limitations

The participants were made to reply the questionnaire before they were discharged which might possibly restrain the patients from speaking their mind because of dependence of care. The fact that quantitative approach was used for measuring patient satisfaction might not fully portray the ideas of the patients which might be complemented using qualitative approach.

## Conclusion

The study concluded that, the overall satisfaction of patients with peri-operative anaesthetic care is 68.8% in which the information provision has been found to be the weakest dimension. It underscores the importance of information and communication as well as staff-patient relationship on patient satisfaction. Meanwhile, better staff-patient relationship and low fear and concern were observed among patients. The results also confirmed the importance of socio-demographic and clinical variables of patients. Improvement can be enhanced by broader and easily understandable information to the public especially in rural areas. Moreover, areas which need to improve include adequacy of information provision to the patients especially during the preoperative period and communication skills of the anaesthetist.

## Additional files

Additional file 1: Patient characteristics (PDF 16 kb)

## Data Availability

The datasets generated and/or analyzed during the current study are available from the corresponding author on a reasonable request.

## Abbreviations

ANOVA: Analysis of Variance
ENT: Ear Nose and Throat
Gyn/Obs: Gynecology and Obstetrics
LPPSq: Leiden Perioperative Care Patient Satisfaction questionnaire
LSD: Least Significant Difference
