# Supplementary material for: Patient satisfaction with peri-operative anesthesia care and associated factors at two National Referral Hospitals: a cross sectional study in Eritrea
Source: BMC Health Serv Res. 2019 Sep 18;19:669. doi: 10.1186/s12913-019-4499-x (PMC6749663; doi:10.1186/s12913-019-4499-x)
Supplement: Supplementary file 1 — Patient characteristics (PDF 16 kb) [file 12913_2019_4499_MOESM1_ESM.pdf]

Additional file 1

Demographic and clinical Characteristics of the participants

**Variables**		**Frequency**	**Percentage**
**Gender**			
	Male	259	55.1
	Female	211	44.9
**Residence**			
	Urban	274	58.3
	Rural	196	41.7
**Occupation**			
	Employed	235	50
	Unemployed	235	50
**Hospital Setting**			
	Halibet	173	36.8
	Orotta	297	63.2
**Health coverage**			
	Paying	358	76.2
	Free	112	23.8
**Type of Anesthesia**			
	General	267	56.8
	Regional	203	43.2
**Type of Surgery**			
	General	261	55.5
	Orthopedic	99	21.1
	Gyn/Obs	89	18.9
	ENT	7	1.5
	Burn	14	3.0
**Case**			
	Emergency	109	23.2
	Elective	361	76.8
**Age**	**Mean**	**SD**	
	45.87	18.53	
